# DNA cross-link repair deficiency enhances human cell sensitivity to colibactin-induced genotoxicity

**DOI:** 10.1186/s41021-025-00339-7

**Published:** 2025-09-22

**Authors:** Masanobu Kawanishi, Osamu Tsubohira, Ai Ueshima, Yuuta Hisatomi, Yoshimitsu Oda, Michio Sato, Noriyuki Miyoshi, Michihiro Mutoh, Hideki Ishikawa, Keiji Wakabayashi, Takashi Yagi, Kenji Watanabe

**Affiliations:** 1 Graduate School of Science, Osaka Metropolitan University, 1-2 Gakuen-cho, Naka-ku, Sakai-shi, 599-8570 Osaka Japan; 2 Department of Pharmaceutical Sciences, University of Shizuoka, Shizuoka, Japan; 3 Graduate Division of Nutritional and Environmental Sciences, University of Shizuoka, Shizuoka, Japan; 4 Department of Molecular-Targeting Prevention, Kyoto Prefectural University of Medicine, Kyoto, Japan

**Keywords:** Colibactin, DNA interstrand crosslinks, Genotoxicity, DNA repair

## Abstract

**Introduction:**

Colibactin is a small genotoxic molecule of polyketide produced by a subset of enteric bacteria including certain *Escherichia coli* (*E. coli*) harbored in the human gut microbiota. Its biosynthesis is governed by a multistep enzymatic process encoded by the polyketide synthase (*pk*s) gene cluster. Colibactin is thought to exert its carcinogenic potential primarily through the induction of DNA interstrand crosslinks (ICLs); however, the precise mechanisms underlying its genotoxicity remain largely unresolved. In this study, we focused on ICL formation and its associated repair pathways to investigate whether colibactin-induced ICLs play a central role in the induction of chromosomal aberrations and inhibition of cell proliferation.

**Findings:**

HAP1 cells deficient in *FANCD2*, a gene essential for ICL repair, and their wild-type counterparts were infected with colibactin producing (*clb*⁺) *E. coli* strains isolated from a Japanese colorectal cancer (CRC) patient. Following recovery culture, the frequency of micronucleated (MN) cells was assessed. The results showed that *FANCD2*-deficient cells exhibited a significantly higher frequency of MN cells compared to wild-type cells. Additionally, the cytotoxicity of the *clb*⁺ strains was evaluated using the XTT assay. *FANCD2*-deficient cells demonstrated higher sensitivity to the *clb*⁺ *E. coli* strains than wild-type cells.

**Conclusion:**

These findings suggest that colibactin, produced by *clb*⁺ *E. coli*, can play a role in the formation of ICLs, thereby contributing significantly to the induction of chromosomal aberrations and the inhibition of human cell proliferation.

## Introduction

Colibactin is a structurally complex, genotoxic small molecule produced by certain strains of *Escherichia coli* (*E. coli*) and other members of the family *Enterobacteriaceae* that inhabit the human gastrointestinal tract. Over the past two decades, increasing evidence has implicated colibactin in the pathogenesis of colorectal cancer (CRC), suggesting a potential role for microbial genotoxins in tumorigenesis. Colibactin-producing (*clb*⁺) bacteria carry a 54-kilobase genomic island known as the *pks* (polyketide synthase) island, which encodes a hybrid nonribosomal peptide synthetase-polyketide synthase (NRPS-PKS) biosynthetic machinery responsible for the synthesis of this genotoxin [1]. The biosynthetic products of this gene cluster include unstable, DNA-reactive intermediates capable of inducing severe DNA damage in host cells. Pioneering work by Nougayrède et al. first demonstrated that infection with *clb*⁺ *E. coli* strains results in DNA double-strand breaks (DSBs) and interstrand cross-links (ICLs) in human epithelial cell lines and murine models, thereby initiating genomic instability and promoting mutagenesis [1]. Subsequent studies have shown that these *clb*⁺ strains are more prevalent in the gut microbiota of patients with inflammatory bowel disease, familial adenomatous polyposis, and CRC, implicating colibactin as a microbiome-derived carcinogenic agent under chronic inflammatory conditions [2, 3]. In addition to experimental evidence, recent large-scale genomic analyses have identified specific mutational signatures in human colorectal tumors that are attributable to colibactin-induced DNA damages, further supporting a causative link between colibactin exposure and colorectal carcinogenesis [4].

Our previous work contributed to this growing body of evidence by demonstrating that *E. coli* strains isolated from a Japanese CRC patient harbor the *pks* island, produce colibactin, and induce genotoxic effects in vitro, including inductions of DNA damage and micronucleus (MN) in infected cells [5, 6]. Despite these advances, the precise molecular mechanisms underlying colibactin-induced genotoxicity, particularly the contribution of ICL formation and the host DNA damage response, remain incompletely understood. In particular, it is unclear to what extent ICLs, as opposed to other forms of DNA lesions, are responsible for genotoxicity following exposure to colibactin.

In the present study, we aimed to elucidate the contribution of colibactin-induced ICLs to genotoxicity using *pks*⁺ *E. coli* strains isolated from a Japanese CRC patient. Specifically, we focused on the ICLs and the involvement of key DNA repair pathway, such as the Fanconi anemia (FA) pathway, to determine whether these lesions play a central role in mediating the genotoxic effects of colibactin. Understanding these mechanisms is essential for clarifying the etiological role of colibactin in CRC and may inform the development of microbiota-targeted prevention or therapeutic strategies.

## Materials and methods

### E. coli strains

The isolation *clb*^+^ *E. coli* strains were previously described in our earlier report [5]. Briefly, strains #50 and #253 were isolated from the T1 region of surgically resected CRC tissue obtained from a 79-year-old male patient. The adenocarcinoma had a diameter of 9.5 cm and was present in the ascending colon. The isogenic *clbP* mutant #50*clbP*^−^ was used as a *clb*^−^ strain. This #50*clbP*^−^ strain was established by deletion of the *clbP* gene (encoding periplasmic peptidase) in *E. coli* #50 with homologous recombination as described previously [5]. The JCM1649T strain was obtained from the Japan Collection of Microorganisms, maintained by the Microbe Division of the RIKEN BioResource Research Center (Tsukuba, Japan), which participates in the National BioResource Project supported by the Ministry of Education, Culture, Sports, Science and Technology (MEXT), Japan. The JCM1649T was also used as a *clb*^−^ strain [7].

### Infection and in vitro micronucleus test

Human myeloid leukemia cell line HAP1 and *FANCD2*-knockout HAP1 cell line [8] were kindly provided by Prof. Minoru Takata (Kyoto Univ.). Bacterial infection to the leukemia cells and the MN test for genotoxicity evaluation [9] were carried out as previously described [6]. In brief, these cell lines were seeded into 60-mm plastic cell culture dishes (5 × 10⁵ cells per dish in IMDM (Nacalai Tesque, Kyoto, Japan) with 10% fetal bovine serum (FBS; Sigma-Aldrich, MO, USA) one day prior to bacterial infection. Bacterial cultures were grown at 37 °C in Infection Medium (IM), consisting of DMEM (Nacalai Tesque, Kyoto, Japan) supplemented with 25 mM HEPES and 5% FBS, until reaching an optical density of OD₅₉₅ = 0.5. Infection was performed by adding 3 mL of IM containing *E. coli* at the indicated multiplicity of infection (MOI), defined as the number of bacteria per host cell at the onset of infection. Following a 4-hour exposure to the bacteria, cells were cultured for an additional 48 h in IMDM with 10% FBS supplemented with 200 µg/mL gentamicin (Nacalai Tesque) at 37 °C in a 5% CO₂ atmosphere. Cell number was measured to evaluate growth inhibition. As a positive control of ICL induction, the cells were treated with mitomycin C (MMC) (FujiFilm Wako Chemicals, Osaka, Japan) for 4 h. Subsequently, the micronucleus (MN) assay was conducted, and the number of cells containing MN was determined by examining 1,000 interphase cells. With Microsoft Excel statistical significance was assessed using a t-test (*p* < 0.05).

### Cytotoxicity assay

HAP1 and *FANCD2*-knockout HAP1 cells were seeded at a density of 5 × 10³ cells per well in a 96-well plate one day prior to the infection. As described above, IM (100 µL) containing *E. coli* at various MOIs was added to each well. The plates were incubated for 4 h at 37 °C in a 5% CO₂ atmosphere. Since *E. coli* could also reduce 2,3-bis-(2-methoxy-4-nitro-5-sulfophenyl)−2*H*-tetrazolium-5-carboxanilide (XTT), to subtract this background activity, 100 µL of the same *E. coli*-containing IM was added to wells of a separate plate without seeded human cells and incubated under identical conditions. After 4 h, the IM was removed, and the wells were washed three times with PBS. Subsequently, 100 µL of IMDM with 10% FBS containing gentamicin was added to each well, followed by incubation for 72 h at 37 °C in a 5% CO₂ atmosphere. After incubation, 100 µL of pre-mixed XTT reaction solution of XTT Cell Proliferation Assay Kit (Funakoshi Co., Ltd., Tokyo, Japan) was added to each well. The plate was gently tapped by hand for 1 min to ensure mixing, and then incubated for approximately 4 h. Absorbance at 450 nm (Abs_450_) was measured using a plate reader (iMark microplate reader, Bio-Rad Laboratories, Inc., CA, USA.). Cell viability was calculated by subtracting the Abs_450_ of the *E. col*i-only wells from the Abs_450_ of transfected well, and then normalizing to the Abs_450_ of the solvent control (treated with IM only). Statistical analysis was performed as mentioned above.

## Results and discussion

### In vitro genotoxicity analysis

**Fig. 1:**
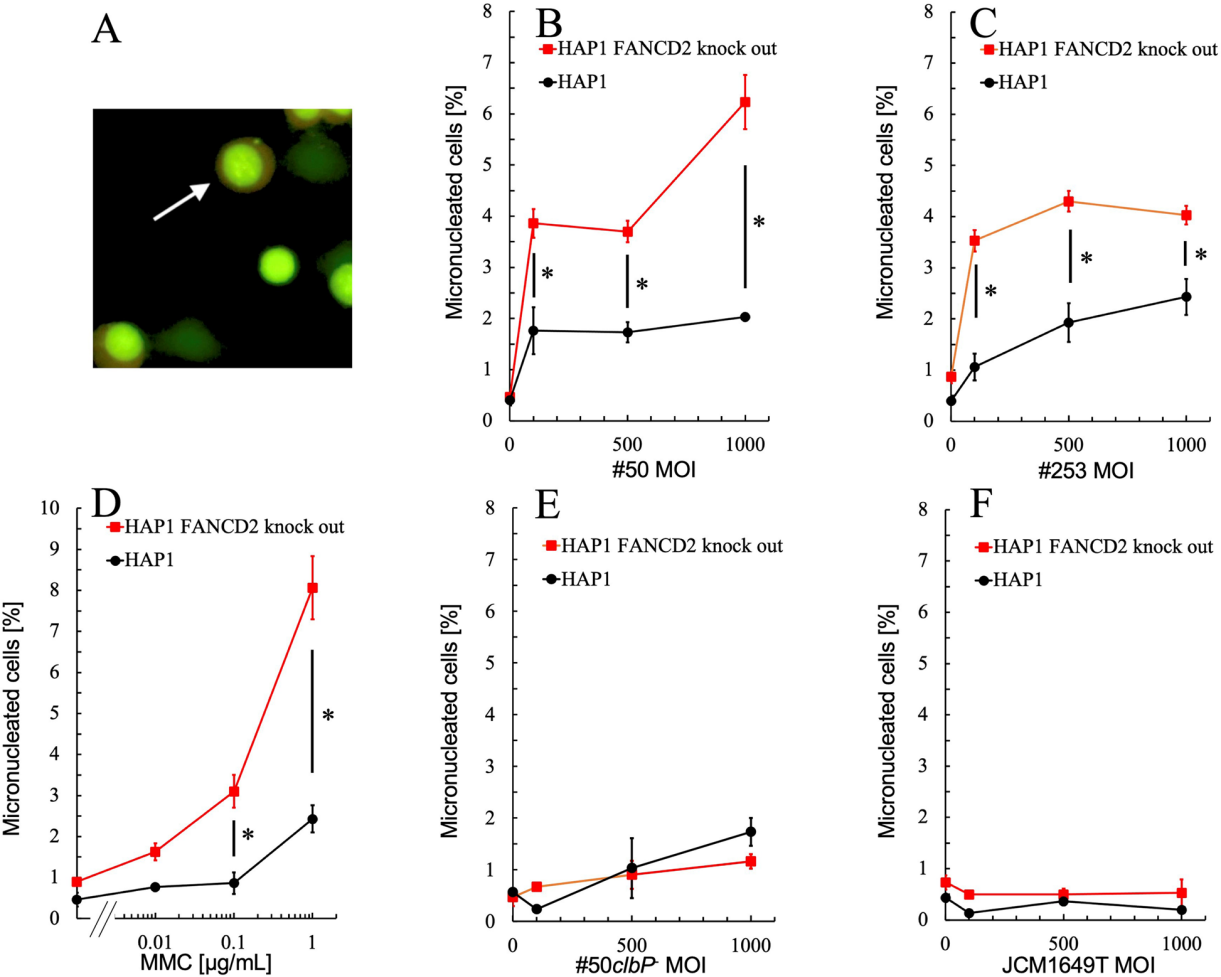
Micronuclei formation in HAP1 *FANCD2*-knockout cells infected with *clb*^*+*^ *E. coli. *MN frequencies (mean ± SD values) at least 1,000 cells are shown. In the graph, MOI = 0 represents the vehicle control (treatment with IM). **A** a representative image of micronucleated HAP1 cells (indicated by white arrow). **B** MN induction by *clb*⁺ strain #50. **C** MN induction by *clb*⁺ strain #253. **D** MN induction by ICL inducing agent MMC. **E** MN induction by *clb*^−^ strain #50*clbP*^−^. F, MN induction by *clb*^−^ strain JCM1649T. * indicates *p* < 0.05 (HAP1 FANCD2-knockout cells versus HAP1 cells at indicated dose) according to Student’s t-test

Colibactin contains two electrophilic cyclopropane moieties that enable covalent bonding with DNA, leading to the formation of ICL [10, 11]. In mammalian cells, ICLs are primarily repaired via the FA pathway, in which the FANCD2 protein plays a central role [12]. Our previous work demonstrated that *clb*⁺ *E. coli* strains #50 and #253, isolated from a Japanese patient with CRC, induced MN in CHO cells, indicative of chromosomal instability [6]. In the present study, we aimed to further investigate the genotoxic effects of these *E. coli* strains in human HAP1 cells (Fig. 1A), with a particular focus on the role of FANCD2 in ICL repair.

Both *clb*⁺ strains (#50 and #253) induced MN formation in HAP1 cells in a MOI-dependent manner or a saturating manner at MOIs above 100 (Fig. 1BC). The MN frequency in solvent control ranged from 0.4 to 0.8% in HAP1 and *FANCD2*-knockout cells. HAP1 cells infected with strain #50, MN frequency increased, reaching 2.0% at MOI 1,000. Notably, in HAP1 *FANCD2*-deficient cells, MN frequency was further elevated, reaching 6.2% at MOI 1,000. Across all MOI conditions tested, HAP1 *FANCD2*-deficient cells exhibited significantly higher MN frequencies compared to their wild-type counterpart HAP1 cells (*p* < 0.05) (Fig. 1B). Similarly, infection with strain #253 led to an increase in MN frequency. At MOI of 1,000, MN frequencies in HAP1 and HAP1 *FANCD2*-knockout cells were 2.4% and 4.3%, respectively. Across all MOI conditions tested, HAP1 *FANCD2*-deficient cells consistently exhibited significantly higher MN frequencies compared to their wild-type counterparts (*p* < 0.05) (Fig. 1C).

As a positive control for ICL induction, MMC treatment resulted in a concentration-dependent increase in MN frequency in both cell lines (Fig. 1D). At the highest concentration tested, MN frequencies reached 2.7% in HAP1 cells and 8.1% in HAP1 *FANCD2*-knockout cells. Notably, at MMC concentrations of 0.1 µg/mL and 1.0 µg/mL, MN frequency was significantly higher in HAP1 *FANCD2*-deficient cells compared to wild-type HAP1 cells, confirming their heightened susceptibility to ICL-induced genotoxicity. In contrast, treatment with the *clb*⁻ strains #50*clbP*^−^ or JCM1649T, used as negative controls, induced slight or no increase in MN frequency in either cell line, respectively (Fig. 1EF). These findings suggest that colibactin produced by *clb*⁺ bacteria could induce ICLs in the genome DNA of human cells, leading to the accumulation of chromosomal aberrations, particularly in cells deficient in effective ICL repair. Interestingly, MN frequencies in wild-type HAP1 cells were generally lower than those previously observed in wild-type CHO cells (e.g., ~ 20% at MOI 1,000 with strain #50) [6], indicating that the extent of genotoxicity may vary depending on the cell type as well. In addition, growth inhibition (versus solvent controls) exceeded 60% in the following conditions: wild-type HAP1 cells treated with strain #50 at MOI ≥ 500 or strain #253 at MOI = 1,000, and *FANCD2*-knockout HAP1 cells treated with strain #50 at MOI ≥ 100, strain #50*clbP*^−^ at MOI = 1,000, or MMC at concentrations ≥ 0.1 µg/mL. Therefore, further experiments are required for an accurate genotoxicity risk assessment.

### Cytotoxicity assay

**Fig. 2:**
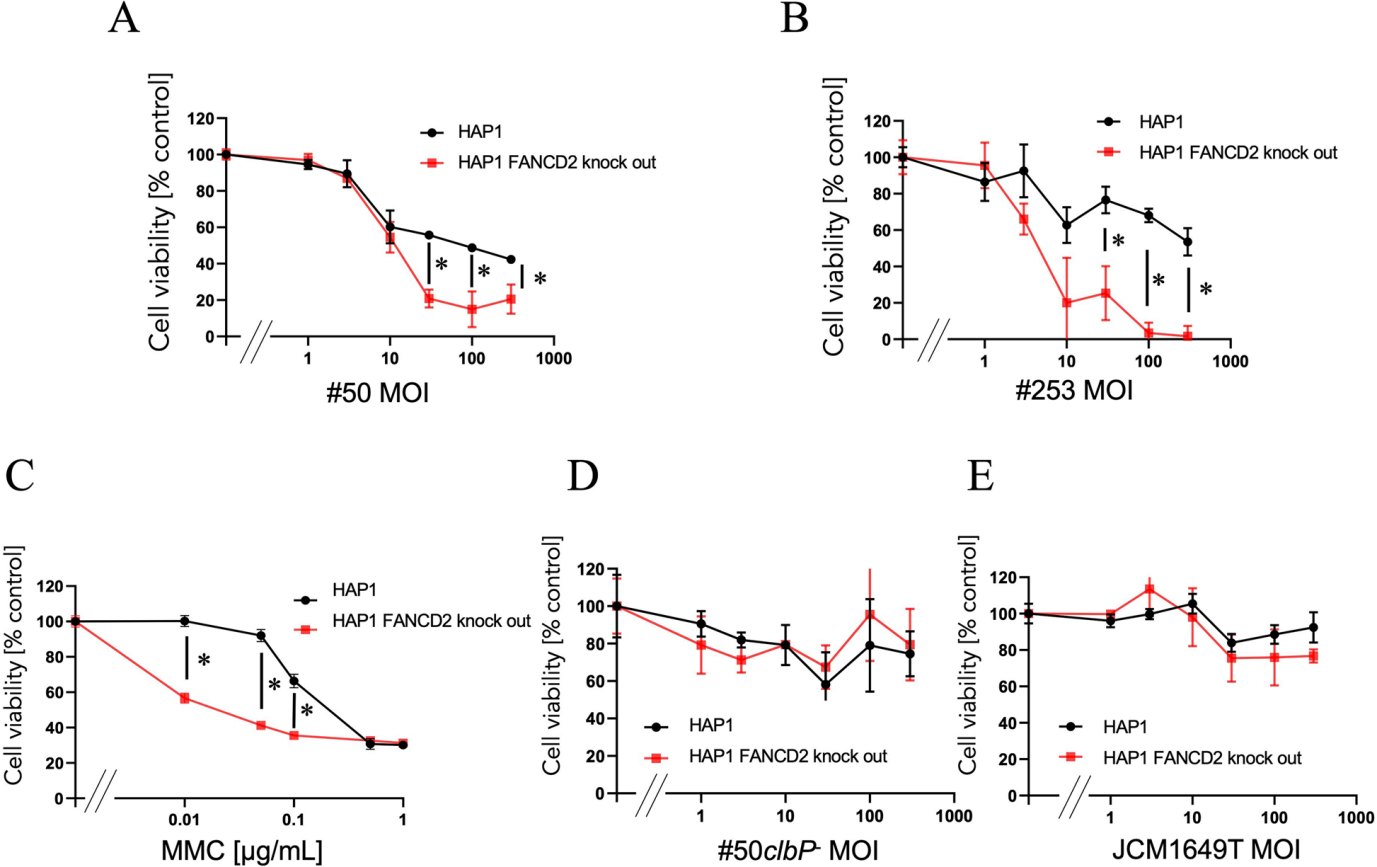
Cell survival of HAP1 *FANCD2*-knockout cells after infection with *clb*^*+*^ *E. coli. *Cell viability (mean ± SD values) at least three independent experiments are shown. In the graph, MOI = 0 represents the vehicle control (treatment with IM). **A** cell viability after infection of *clb*⁺ strain #50. **B** cell viability after infection of *clb*⁺ strain #253. **C** cell viability after treatment of ICL inducing agent MMC. **D** cell viability after infection of *clb*^−^ strain #50*clbP*^−^. **E** cell viability after infection of *clb*^−^ strain JCM1649T. * indicates *p* < 0.05 (HAP1 FANCD2-knockout cells versus HAP1 cells at indicated dose) according to Student’s t-test

To further evaluate the impact of colibactin on cellular viability, we conducted cytotoxicity assays based on cell survival following bacterial infection (Fig. 2). Infection with strain #50 resulted in an MOI-dependent decrease in viability in both wild-type and *FANCD2*-deficient HAP1 cells (Fig. 2A). At MOI 300, the cell viability of HAP1 and HAP1 *FANCD2*-knockout cells were 42% and 21%, respectively. Statistically significant differences in viability were observed at MOIs of 30 and above (*p* < 0.05), with *FANCD2*-deficient cells showing greater sensitivity. A similar trend was observed with strain #253. At MOI 300, viability was 54% in wild-type cells but plummeted to just 1.6% in *FANCD2*-deficient cells (*p* < 0.05) (Fig. 2B). In contrast, treatment with *clb*⁻ #50*clbP*^−^ and JCM1649T resulted in comparable survival between the two cell lines, even at higher levels of bacterial infection, no substantial decrease in cell viability was observed (Fig. 2DE). Additionally, HAP1 *FANCD2*-knockout cells exhibited heightened sensitivity to MMC treatment (*p* < 0.05) (Fig. 2C). These findings could reinforce the notion that colibactin-induced cytotoxicity is primarily mediated through the formation of ICLs, and that FANCD2 plays a critical protective role. The heightened sensitivity of *FANCD2*-deficient cells to both *clb*⁺ bacterial infection and MMC exposure underscores the importance of this repair pathway in mitigating colibactin-associated DNA damage.

In the present study, however, the amount of DNA damage was not quantified. Therefore, it is important to note that we cannot exclude the possibility that the extent of DNA damage induced by infection with *clb*⁺ strains varied among different cell types.

## Conclusion

Our results demonstrate that human cells deficient in FANCD2, a key component of ICL repair pathway, are significantly more susceptible to genotoxic and cytotoxic effects induced by *clb*⁺ *E. coli*. Both MN formation and loss of cell viability were markedly exacerbated in *FANCD2*-deficient HAP1 cells compared to wild-type counterpart cells. These findings could suggest that colibactin may induce ICLs in genomic DNA, potentially leading to chromosomal instability and impaired cell proliferation, particularly in the absence of efficient ICL repair mechanisms. This study provides further mechanistic insight into how colibactin contributes to genomic instability and supports its proposed role in the etiology of CRC.

## Data Availability

No datasets were generated or analysed during the current study.

## Abbreviations

CHO: Chinese hamster ovary
*clb*^+^: colibactin-producing
CRC: Colorectal cancer
FA: Fanconi anemia
FANCD2: Fanconi anemia complementation group D2
ICL: DNA interstrand crosslink
MN: Micronucleus
MOI: Multiplicity of infection
pks: Polyketide synthase
